# Family Systems and Cognitive Health: Experiences of Black Midlife and Older Adults in South Carolina

**DOI:** 10.1093/geroni/igaf122.1177

**Published:** 2025-12-31

**Authors:** Abigail Stephan, Lena Simon, Angie Sardina, Jeff Meadows, Marsha Hampton, Lesley Ross, Alyssa Gamaldo

**Affiliations:** Clemson University, Clemson, South Carolina, United States; Clemson University, Clemson, South Carolina, United States; Clemson University, Clemson, South Carolina, United States; Clemson University Institute for Engaged Aging, Clemson, South Carolina, United States; Clemson University, Clemson, South Carolina, United States; Clemson University, Clemson, South Carolina, United States; Clemson University, Clemson, South Carolina, United States

## Abstract

The family is one of the most impactful contexts for development across the lifespan. Systems theories posit that families can act as sources of support or strain that impact individual outcomes, while individual members’ health and well-being simultaneously have meaningful effects on entire family units. This is exceedingly true for families navigating—or preparing for—changes in older members’ cognitive function. At the same time, families are embedded within larger systems that influence beliefs and behaviors, therefore requiring consideration of the broader context of the social determinants at play when families engage in cognitive healthcare planning. Accordingly, the current investigation highlights the voices of Black midlife and older adults within family systems—consisting of both relatives and fictive kin—from rural and suburban communities in South Carolina who participated in a focus group about their families’ approaches to discussing and making decisions on matters pertaining to aging, such as Alzheimer’s disease and related dementias (ADRD). Our team conducted seven focus groups (three in rural and four in suburban communities) with 5-7 participants in each group (N = 40, M = 70.25 years, 45-89 years, 97.5% Black, 82.5% female). Preliminary findings from our thematic analysis highlight similarities (e.g., salience of personal experiences) and differences (e.g., beliefs about support structures and obligations) in families’ perspectives of ADRD planning by rural v. suburban locale. These findings support the importance of taking a nuanced, integrated approach when considering the role played by family and place, both with their own historical, structural, and social complexities, in cognitive healthcare.

